# An evaluation of service user experience, clinical outcomes and service use associated with urgent care services that utilise telephone-based digital triage: a systematic review protocol

**DOI:** 10.1186/s13643-021-01576-x

**Published:** 2021-01-13

**Authors:** Vanashree Sexton, Jeremy Dale, Helen Atherton

**Affiliations:** Unit of Academic Primary Care, Warwick Medical School, University of Warwick, Gibbet Hill, Coventry, CV7 4AL UK

**Keywords:** Digital interventions, Triage, Primary care, Urgent care, Emergency care, Telephone triage, Narrative synthesis

## Abstract

**Background:**

Telephone-based digital triage is widely used by services that provide urgent care. This involves a call handler or clinician using a digital triage tool to generate algorithm-based care advice, based on a patient’s symptoms. Advice typically takes the form of signposting within defined levels of urgency to specific services or self-care advice. Despite wide adoption, there is limited evaluation of its impact on service user experience, service use and clinical outcomes; no previous systematic reviews have focussed on services that utilise digital triage, and its impact on these outcome areas within urgent care. This review aims to address this need, particularly now that telephone-based digital triage is well established in healthcare delivery.

**Methods:**

Studies assessing the impact of telephone-based digital triage on service user experience, health care service use and clinical outcomes will be identified through searches conducted in Medline, Embase, Cumulative Index to Nursing and Allied Health Literature (CINAHL), Web of Science and Scopus. Search terms using words relating to digital triage and urgent care settings (excluding in-hours general practice) will be used. The review will include all original study types including qualitative, quantitative and mixed methods studies; studies published in the last 20 years and studies published in English. Quality assessment of studies will be conducted using the Mixed Methods Appraisal Tool (MMAT); a narrative synthesis approach will be used to analyse and summarise findings.

**Discussion:**

This is the first systematic review to evaluate service user experience, service use and clinical outcomes related to the use of telephone-based digital triage in urgent care settings. It will evaluate evidence from studies of wide-ranging designs. The narrative synthesis approach will enable the integration of findings to provide new insights on service delivery. Models of urgent care continue to evolve rapidly, with the emergence of self-triage tools and national help lines. Findings from this review will be presented in a practical format that can feed into the design of digital triage tools, future service design and healthcare policy.

**Systematic review registration:**

This systematic review is registered on the international database of prospectively registered systematic reviews in health and social care (PROSPERO 2020 CRD42020178500).

**Supplementary Information:**

The online version contains supplementary material available at 10.1186/s13643-021-01576-x.

## Background

Telephone-based digital triage has been widely used by services that provide urgent care over the last several decades [1, 2]. Urgent care is the ‘the range of responses that health and care services provide to people who require – or who perceive the need for – urgent advice, treatment or diagnosis’ [3]. Within urgent care, different types of services utilise telephone-based digital triage, including national or regional help-lines, out-of-hours centres and emergency care providers. Examples of telephone-based services include England’s National Health Service (NHS) 111 service, Scotland’s NHS 24 service, Denmark’s medical help line (MH1813), Australia’s HealthDirect and the MayoClinic telephone service based in the USA [4–9].

Digital triage within these services involves a care service staff member using a digital triage tool to generate algorithm-based care advice, based on a patient’s symptoms. Advice typically takes the form of signposting within defined levels of urgency to specific services, such as an emergency department (ED), out-of-hours centre, general practice (GP) appointment or self-care advice.

In part, these services have been implemented in response to increasing demand on primary care and hospital-based EDs over the last several decades [10]. They offer the potential to manage demand and improve consistency of care by providing a clear entry point or ‘front door’ to patients seeking care [11], which may simplify the patients decision on which service to access [12], and by providing appropriate advice based on the patient’s symptom assessment [13]. There is a need for an up-to-date evaluation of the impact of these services on user experience, service use and clinical outcomes following triage, in order to evaluate success of these services and identify areas for improvement or further research.

Systematic reviews in this research area were conducted several years ago (between 2005 and 2012) [1, 10, 14–16]. Whilst their findings are useful in guiding research, in many cases, they have limited relevance as a result of the reorganisation of services in recent years [2]; an example of reorganisation is England’s introduction of NHS 111 in 2011 [17], involving a workforce shift [18] away from the previous nurse led model to a non-clinician-led service [11]; this demonstrates the need to review more recent studies conducted within these services.

Despite wide adoption of digital triage within urgent care, previous reviews have not focussed on the digital triage element of services. In older literature, digital triage is often referred to as the use of computerised ‘clinical decision support systems’ (CDSS) in the context of telephone triage or consultation, as they were previously known [15]. Instead of focussing on digital triage, previous systematic reviews addressed broader review questions to evaluate telephone triage, including services that use digital triage and those that are not digitally supported [1, 10, 14] or evaluate the use of CDSS on patient outcomes in wider healthcare functions, ranging from digital triage within primary care to treatment management in intensive care units [15].

These previous reviews show mixed results in terms of service user experience, clinical and service use outcomes, which likely result from varying contextual factors, including whether services use digital triage, the type of service, care setting, levels of clinical supervision, types of staff conducting triage and level of staff training. Compared to previous reviews, this review addresses a more narrow review question, which is focussed on services that utilise digital triage in the provision of out-of-hours urgent care. We are excluding ‘in hours’ care as to date digital triage has not been widely adopted in these settings during usual business opening hours.

This review additionally addresses the need to evaluate more recent studies that have analysed large routine triage and patient outcomes datasets that have become more readily available in recent years [11]. Previous reviews included studies with quantitative designs only [10, 14, 15]; this review will additionally include studies exploring patient outcomes through qualitative or mixed methods approaches [17] and will therefore facilitate the integration of findings from studies with mixed designs. Integration will allow for better understanding of the impact of digital triage on service user and patient outcomes, which may provide insights for the future development of digital triage and policy related to such service developments. Findings could also feed into the design of the newly emerging patient self-triage approaches that are being adopted by care services [19, 20], for example the NHS 111Online, which allows patients to self-triage and receive care advice online [21].

### Review question

How does telephone-based digital triage affect service user experience, clinical outcomes and health care service use in patients using out-of-hours urgent care services?

### Objectives

This review will explore the objectives below in out-of-hours urgent care services that utilise telephone-based digital triage:

1. To describe characteristics of patients accessing these services and the triage advice they receive
2. To explore service user (patient or carer) experience of triage
3. To evaluate patient health care service use following triage, including hospital admissions, ED attendance and GP attendance.
4. To evaluate patient clinical outcomes, including hospitalisations and mortality

## Methods

A completed Preferred Reporting Items for Systematic Review and Meta-Analysis Protocols (PRISMA-P) checklist [22] showing the recommended items to include in a systematic review is included in Additional file 1.

### Eligibility criteria

Eligibility criteria have been developed using the population, interventions, comparators, outcomes and study designs (PICOS) principle [23] and will be applied to studies that are included in the review.

#### Population

The review will include studies that evaluate the use of triage in the general population or within particular sub-groups of the general population (for example children or older people).

#### Interventions

The following eligibility criteria relating to the digital triage intervention will be applied to include:

1. Studies that assess the use of telephone-based digital triage in out-of-hours services that provide urgent care; these may include national or regional call centre-based urgent care telephone services, out-of-hours and urgent care centres and ambulance services. Services that only operate during ‘in-hours’ (for example, the use of digital triage for same day GP appointments) will not be included
2. Studies assessing the use of digital triage by the general population for any symptoms (not condition specific)
3. Studies assessing the use of digital triage that results in signposting (advising the patient to attend a local care service, such as an ED, an out-of-hours centre or advising the patient to book a GP appointment) and/or providing self-care advice

#### Outcomes

Studies that assess outcomes relating to at least one of the following outcomes will be included:

1. Characteristics of patients and triage advice
2. Service user (patient or carer) experiences
3. Health care service use following triage: including hospital admissions, ED attendance and GP attendance
4. Patient clinical outcomes, including hospitalisations (number of hospitalisations, duration of hospitalisation, type of hospitalisation) and mortality

#### Study designs

All study types will be included: qualitative (interviews, focus groups, ethnography), quantitative (cohort studies, cross-sectional studies, randomised controlled trials) and mixed methods studies.

Additionally, only studies published in the English Language in the last 20 years will be included (studies conducted from 2000 to 2020): this time period has been chosen to identify changes in outcomes over time in relation to changing models of service delivery, for example changes in workforce mix [2, 18]. Full inclusion and exclusion criteria can be found in Appendix 1.

### Search strategy

Research databases will be searched using a search strategy and key words that have been developed with input from a librarian.

Search terms will include variations of terms relating to ‘urgent care’, ‘triage’ and ‘digital’. Full search terms can be found in Appendix 2. A search will be conducted using the key words and Boolean strategies of ‘AND’ and ‘OR’. The search terms will be modified as necessary according to the database being searched. The following databases will be searched: Medline (Ovid SP), Embase (Ovid SP), CINAHL, Web of Science and Scopus.

The search will be restricted to include studies published between the years 2000 and 2020, studies published in English, and studies electronically published (Epub) ahead of print.

### Data management and screening

References identified in the searches will be managed in Covidence systematic review management software; identified references will be imported into Covidence and de-duplicated.

In the first screening stage, titles and abstracts will be screened against the inclusion and exclusion criteria by two reviewers independently. References that meet the inclusion criteria will be screened again for inclusion at full-text level, by two reviewers independently. For any full-text articles that are excluded, exclusion reasons will be documented using Covidence.

Any discrepancies on studies to be included at both screening stages will be resolved through discussion between the two reviewers. If a consensus is not reached, a third reviewer will be consulted. At the end of the two screening stages, a final set of studies to be included will be identified. The study selection process will be described through a Preferred Reporting Items for Systematic Reviews and Meta-Analyses (PRISMA) flow chart [24].

Reviewers will independently extract relevant data from the included studies which will be recorded on a custom pre-defined data extraction form. The following information will be extracted and entered into the data extraction form: author, publication year, country, study design, care setting, participants, intervention details, type of care service staff conducting triage (doctor/nurse/paramedic/non-clinician), comparator, outcomes, effect of intervention and contextual factors (for example: staff experience and training, time that the service has been in place, level of support available to call takers). Data extraction discrepancies will be resolved through discussion between the reviewers, and a third reviewer will be consulted if necessary. Study authors may be contacted during the screening or data extraction where eligibility is unclear.

References of included studies will be screened by hand to identify any other eligible studies. Different reports that relate to the same study will be identified and labelled to indicate that they refer to the same study.

### Risk of bias and quality assessment

Quality assessment will be conducted for all full-text peer-reviewed publications that fit the inclusion criteria, using the Mixed Methods Appraisal Tool version 2018 (MMAT) [25], which is designed to enable the assessment of mixed studies.

If the reviewers disagree in their assessment of bias in a study, this will be resolved though discussion. Quality assessment will not be used to exclude studies from the review but will be taken into account in the synthesised findings.

Different types of biases which may be present in each study will be considered and presented in a risk of bias table. If missing data or selective reporting of outcomes is apparent in a study, the study author will be contacted to obtain information on the reasons behind the missing data and to assess the risk of any systematic differences in missing data. Studies of equal quality as determined through assessment with the MMAT and risk of bias assessment will be considered to have similar weighting, and this will feed into the data synthesis to ensure trustworthiness of synthesis, serving to minimise bias.

Additionally, for quantitative studies, the occurrence of reporting (non-publication) bias will be evaluated by conducting checks of study registers (for example: ClinicalTrials.gov) to identify the completeness of the published literature included in the review; these findings will feed into the overall evaluation of the available evidence.

### Strategy for data synthesis

A narrative synthesis approach will be used, which is a ‘synthesis of findings from multiple studies that relies primarily on the use of words and text to summarise and explain the findings’ [26]. This strategy has been chosen as the included studies are likely to be diverse in design and outcomes.

Narrative synthesis will be conducted to analyse, integrate and summarise the evidence identified through data extraction and the findings from quality assessment. An iterative approach will be followed, based on four main elements: (1) theory development, (2) preliminary synthesis, (3) exploring relationships between evidence from studies and (4) assessing robustness of the synthesis conducted [26]. Key sub-groups and subsets of data will be identified through narrative synthesis, based on the findings of the included studies.

## Discussion

This review seeks to evaluate the impact of telephone-based digital triage by urgent care services on service user experience, and patients’ clinical and service use outcomes. This is the first systematic review to evaluate these outcomes in relation to digital triage in the urgent care setting. In addition, this review includes mixed studies, enabling the integration of evidence from studies of wide-ranging design. It will be possible to investigate how findings have changed over time, by comparing results of studies carried out early in the implementation of these services as well more recent studies conducted in well-established services, and how other contextual factors influence findings. Urgent care delivery continues to develop rapidly; findings from this review will have potential to inform policy and practice related to the design and delivery of urgent care service delivery and should also highlight gaps in the evidence that require further investigation.

### Registration of review

This review is registered on the international database of prospectively registered systematic reviews in health and social care (PROSPERO 2020 CRD42020178500). Amendments to the protocol will be amended on the registration.

### Supplementary Information

**Additional file 1.** PRISMA-P checklist.

## Data Availability

Not applicable.

## Appendix 1

**Table 1:** Inclusion and Exclusion criteria

Inclusion	Exclusion
Studies assessing telephone-based digital triage	Studies assessing telephone triage that is not digitally supported (e.g. triage conducted through paper protocols) Studies assessing digital triage that is not telephone based (face to face)
Studies investigating telephone-based digital that is used for any/broad ranging symptoms (not condition specific)	Studies assessing the use of digital triage for specific conditions (for example, digital tools that provide patient condition self-management or Cognitive Behavioural Therapy would be excluded)
Studies investigating telephone-based digital triage that conducted by a member of health care service staff (clinician or non-clinician)	Studies investigating digital triage that used by a patient directly for self-triage (e.g. 111online)
Studies that examine the use of digital triage tools resulting in signposting and/or self-care advice for the patient: Examples of signposting include advice to the patient to book a GP appointment, attend ED, ambulance dispatch and self-care	Studies that examine the use of digital triage tools resulting in other types of advice (e.g. condition specific advice only)
Telephone-based digital triage in services that provide urgent care, predominantly out of hours, including: Call centre-based urgent care telephone services (examples: NHSDirect, NHS111), which may provide care 24/7 Out-of-hours and urgent care centres Out-of-hours services run by general practices Ambulance services (include only secondary triage of non-emergency calls, following initial assessment)	Studies in routine care settings. Exclude triage services that only provide in-hours digital triage (for example, those used within usual general practice opening hours only). Exclude triage that is utilised by hospital-based emergency departments, for example: the ‘Canadian Triage and Acuity Scale’ and the ‘Manchester Triage System’
Studies assessing outcomes relating to: 1. Patterns of telephone triage service use by patients 2. Service user (patient or carer) experience 3. Service use following triage, including: ED attendance, GP attendance and hospitalisations) 4. Health outcomes following triage, including mortality and hospitalisations	Studies that only explore outcomes that are not in the included list: e.g. Studies that only explore experience of the staff member who uses the digital triage tool (e.g. non-clinician call handler for NHS 111, or nurse call taker for NHS Direct) Accuracy outcomes: relating to comparison of triage outcomes between types of professionals
Studies of any design will be included Examples: qualitative (interviews, focus groups, ethnography), quantitative (cohort studies, cross-sectional studies or RCTs) or mixed methods studies.	Reviews, discussion articles, conference abstracts, case reports
Studies published in English	Studies published in other languages
Studies published in the last 20 years	Studies published prior to 20 years ago

## Appendix 2

### Search terms

**Table 2:** Medline search terms

Concept	Search terms
Care setting	Primary care.mp OR Primary Health Care/ OR After-Hours Care/ OR Out-of-hours.mp OR Emergency care.mp OR Emergency Medical Services/ OR Urgent care.mp OR Ambulatory Care/ or ambulatory care.mp AND
Triage	Triage.mp OR Triage/ OR Telephone consultation.mp AND
Digital	Digital.mp OR Computer.mp OR Software/ or Software.mp OR Online.mp or Online Systems/ OR Internet.mp or Internet/ OR Web.mp or Web Browser/ OR Computerised.mp OR Computerized.mp OR electronic.mp OR ECDS.mp OR CCDS* OR Decision Support Systems, Clinical/ OR Decision support*

**Table 3:** EMBASE search terms

Concept	Search terms
Care setting	Primary care.mp OR Primary Medical Care/ OR After hours Care/ OR Out-of-hours.mp OR out-of-hours care/ OR Emergency care.mp OR Emergency Health service/ OR emergency care/ OR Urgent care.mp OR Ambulatory Care/ OR ambulatory care.mp AND
Triage	Triage.mp OR Telephone consultation.mp OR teleconsultation/ AND
Digital	Digital.mp OR Computer.mp OR Software/ or Software.mp OR Online.mp or Online System/ OR Internet.mp or Internet/ OR Web.mp or Web Browser/ OR Computerised.mp OR Computerized.mp OR electronic.mp OR ECDS.mp OR CCDS* OR Decision Support Systems / OR Decision support.mp

**Table 4:** CINAHL search terms

Concept	Search terms
Care setting	‘Primary care’ OR (MH ‘Primary Health Care’) OR ‘Out-of-hours’ OR ‘After-hours care’ OR (MH ‘Emergency Care’) OR ‘Emergency care’ OR (MH ‘Emergency Service’) OR ‘Urgent care’ OR (MH ‘Ambulatory Care’) OR ‘Ambulatory care’ AND
Triage	(MH ‘Triage’) OR ‘triage’ OR ‘Telephone consultation’ AND
Digital	‘digital’ OR ‘Computer’ OR (MH ‘Software’) OR ‘software’ OR ‘Online’ OR (MH ‘Online Systems’) OR (MH ‘Internet’) OR ‘Internet’ OR ‘web’ OR (MH ‘Web Browsers’) OR ‘Computerised’ OR ‘computerized’ OR ‘electronic’ OR ‘ECDS’ OR ‘CCDS’ OR ‘Decision support’

**Table 5:** Web of Science search terms

Concept	Search terms
Care setting	‘Primary care’ OR ‘Primary Health Care’ OR ‘After-Hours Care’ OR Out-of-hours OR ‘Emergency care’ OR ‘Emergency Medical Services’ OR ‘Urgent care’ OR ‘Ambulatory Care’ AND
Triage	Triage OR ‘Telephone consultation’ AND
Digital	Digital OR Computer OR Software OR Online OR Internet OR Web OR Computerised OR Computerized OR electronic OR ECDSOR CCDS* OR ‘Decision support system’

**Table 6:** Scopus search terms

Concept	Search terms
Care setting	‘Primary care’ OR ‘Primary Health Care’ OR ‘After-Hours Care’ OR ‘Out-of-hours’ OR ‘Emergency care’ OR ‘Emergency Medical Services’ OR ‘Urgent care’ OR ‘Ambulatory Care’ AND
Triage	Triage OR ‘Telephone consultation’ AND
Digital	Digital OR Computer OR Software OR Online or ‘Online Systems’ OR Internet OR Web OR Web Browser OR Computerised OR Computerized OR electronic OR ECDS OR CCDS OR ‘Decision support system’

## Abbreviations

CINAHL: Cumulative Index to Nursing and Allied Health Literature
PRISMA: Preferred Reporting Items for Systematic Reviews and Meta-Analyses
PRISMA-P: Preferred Reporting Items for Systematic Reviews and Meta-Analyses Protocols
GP: General Practice
NHS: National Health Service
CDSS: Clinical decision support system
ED: Emergency department
MMAT: Mixed Methods Appraisal Tool
